# Ac2-26 mitigated acute respiratory distress syndrome rats via formyl peptide receptor pathway

**DOI:** 10.1080/07853890.2021.1925149

**Published:** 2021-05-19

**Authors:** Yingnan Ju, Lin Qiu, Xikun Sun, Hengyu Liu, Wei Gao

**Affiliations:** a Department of ICU, The Cancer Hospital of Harbin Medical University, Harbin, China; b Department of Anesthesia, The Second Affiliated Hospital of Harbin Medical University, Harbin, China

**Keywords:** Ac2-26, acute respiratory distress syndrome, lung injury

## Abstract

**Background:**

Acute respiratory distress syndrome (ARDS) is characterized by severe local and systemic inflammation. Ac2-26, an Annexin A1 Peptide, can reduce the lung injury induced by reperfusion via the inhibition of inflammation. The present study aims to evaluate the effect and mechanism of Ac2-26 in ARDS.

**Methods:**

Thirty-two rats were anaesthetized and randomized into four groups: sham (S), ARDS (A), ARDS/Ac2-26 (AA), and ARDS/Ac2-26/BOC-2 (AAB) groups. Rats in the S group received saline for intratracheal instillation, while rats in the other three groups received endotoxin for intratracheal instillation, in order to prepare the ARDS and inject the saline, Ac2-26, and Ac2-26 combined with BOC-2. After 24 h, the PaO_2_/FiO_2_ ratio was calculated. The lung tissue wet-to-dry weight ratio and the protein level in bronchoalveolar lavage fluid (BALF) were tested. Then, the cytokines in BALF and serum, and the inflammatory cells in BALF were investigated. Afterwards, the oxidative stress response and histological injury was evaluated. Subsequently, the epithelium was cultured and analyzed to estimate the effect of Ac2-26 on apoptosis.

**Results:**

Compared to the S group, all indexes worsened in the A, AA, and AAB groups. Furthermore, compared to the S group, Ac2-26 significantly improved the lung injury and alveolar-capillary permeability, and inhibited the oxidative stress response. In addition, Ac2-26 reduced the local and systemic inflammation through the regulation of pro- and anti-inflammatory cytokines, and the decrease in inflammatory cells in BALF. Moreover, Ac2-26 inhibited the epithelium apoptosis induced by LPS through the modulation of apoptosis-regulated proteins. The protective effect of Ac2-26 on ARDS was partially reversed by the FPR inhibitor, BOC-2.

**Conclusion:**

Ac2-26 reduced the lung injury induced by LPS, promoted alveolar-capillary permeability, ameliorated the local and systemic inflammation, and inhibited the oxidative stress response and apoptosis. The protection of Ac2-26 on ARDS was mainly dependent on the FPR pathway.

## Introduction

Acute respiratory distress syndrome (ARDS) is a common complication of severe local or systemic infection, and is characterized by increased alveolar-vascular permeability and lung edoema, pulmonary local or systemic inflammation, and severe hypoxemia [1]. The prevalence is approximately 30.0, 46.6, and 23.4% for mild, moderate and severe ARDS [2], and the mortality of ARDS remains high (27–45%) [1,3], even though various treatments have been applied in clinic. During the ARDS process, the unbalanced inflammation and over production of oxidant species play a pivotal role [3,4].

Annexin A1 (AnexA1) is a endogenous glucocorticoid-regulated anti-inflammatory [5], and its N-terminal-derived peptide Ac-ANX-A1 (Ac 2-26) has been indicated to reduce lung reperfusion injury secondary to intestinal ischaemia reperfusion [6,7]. Furthermore, the expression of formyl peptide receptor (FPR) in endothelial cells and epithelial cells significantly increases after the stimulation of endotoxins [8], and the protection of Ac2-26 is mainly dependent on the activation of FPR [6,7]. The previous studies conducted by the investigators also indicated that Ac2-26 protected the lungs and brain from ischaemia and reperfusion injury via reducing local inflammation [9,10]. These studies suggest that, as an active peptide of Annexin A1, Ac2-26 has a powerful and effective anti-inflammatory property. Therefore, as an endogenous glucocorticoid-regulated anti-inflammatory protein, it was speculated that Ac2-26 can reduce ARDS in rats partly via the FPR pathway.

## Materials and methods

### Study design

Thirty-two Sprague-Dawley (SD) rats were randomized into four groups: sham (S), ARDS (A), ARDS/Ac2-26 (AA), and ARDS/Ac2-26/BOC (AAB) groups (*n* = 8). All rats received anaesthesia with 3% pentobarbital sodium (30 mg/kg intraperitoneal injection) and intubation. After anaesthesia and local infiltration of lidocaine, the caudal artery and vein were cannulated for arterial blood gas analysis, blood samples were collected, and the saline or drug was injected. Rats in the S group received 0.5 ml of saline for intratracheal instillation, while rats in the A, AA and AAB groups were injected with the intratracheal instillation of *Escherichia coli* LPS of 1 mg/kg, which was diluted to 0.5 ml (serotype 055:B5, Sigma-Aldrich, Israel) to simulate the ARDS. Then, the rats were extubated [11]. After instillation, rats in the S and A groups received saline for intravenous injection, while rats in the AA and AAB groups received Ac2-26 (1 mg/kg [7,9,10]) and Ac2-26/BOC (600 ng/kg [12]), respectively. After 24 h, all rats were anaesthetized, as previously described, and intubated. Then, all rats were ventilated for 15 min, with a tidal volume of 10 ml/kg, a respiratory rate of 50 breaths/min, a inspiratory-to-expiratory ratio of 1:2, an inspired oxygen fraction (FiO_2_) of 50%, and a positive end-expiratory pressure of 5 cmH_2_O. After 15 min of ventilation, all rats were sacrificed with over dose of anaesthetics.

### Sample collection

The peripheral blood and arterial blood analysis were conducted at baseline and at 24 h after LPC instillation. A different part of the right lung tissue was collected for the lung histological injury, apoptosis, and protein expression analysis. The left lung was collected to inject the saline and collect the bronchoalveolar lavage fluid (BALF). Briefly, a total of 10 ml/kg of saline (4 °C) containing thylene-diamine-tetraacetic acid (EDTA)-2Na was injected into the left lung, and withdrawn for five times, in order to collect the BALF. Then, the BALF and serum were centrifuged at 1,000 g for 15 min at 4 °C. Afterwards, the supernatant from the serum and BALF was preserved at −80 °C to further analysis.

### Effect of Ac2-26 on alveolar-capillary permeability

The PaO_2_ was determined by arterial blood gas analysis using the Rapidlab 348 system (Bayer Diagnostics, Germany), and the PaO_2_/FiO_2_ ratio was calculated. The protein concentration in BALF was also determined using the BCA method. Next, the lung tissue was weighed and dried at 60 °C for 48 h, and the wet/dry weight ratio was calculated.

### Effect of Ac2-26 on local and systemic inflammation

The focal and systemic inflammation was evaluated by determining the inflammatory factors in the peripheral blood samples and BALF. The TNF-α, IL-1β, IL-6, and IL-10 in peripheral blood and BALF were detected using ELISA kits (Wuhan Boster Bio-Engineering Limited Company, Wuhan, Hubei, China). Then, the number of macrophages and neutrophils in the BALF deposits was counted through Giemsa staining by an independent pathologist.

### Effect of Ac2-26 on oxidative stress response

A part of the right lung tissue was homogenized with saline to prepare the homogenization. The homogenization was centrifuged, and the supernatant was collected to determine the activity of myeloperoxidase (MPO) and NADPH, and the concentration of MDA using the specific kits (Nanjing Jiancheng, Nanjing, China).

### Effect of Ac2-26 on histopathologic lung injury

The lung tissue histological injury was evaluated through haematoxylin and eosin (H&E) staining by a pathologist. Briefly, part of the right lung middle lobe was collected, and the lung tissue was fixed with paraformaldehyde. All lung tissues were embedded in paraffin after dehydration and dealcoholization. The lung tissue in paraffin was cut into 4-μm sections, and these sections were preserved on a slide. After deparaffinization, the slides were stained with H&E. The severity of the lung injury was quantified by two independent investigators who were blinded to the present study. The scores for lung injury is presented in Table 1, which included five variables: lung haemorrhage, peri-bronchial infiltration of inflammatory cells, pulmonary interstitial edoema, pneumocyte hyperplasia, and intra-alveolar infiltration of inflammatory cells. The lung injury score ranged within 0–10 [13].

**Table 1.** Lung injury evaluation variables.

Parameters	Score
Haemorrhage	0 or 1
Peri-bronchial infiltration	0 or 1
Interstitial edoema	0–2
Pneumocyte hyperplasia	0–3
Intra-alveolar infiltration	0–3

### Western blot

In order to observe the expression of various proteins in lung tissues, a part of the lung tissue was collected and the protein was extracted from the lung tissue. Then, the concentration of the protein was determined by Bradford assay. Equivalent protein levels were added to each polyacrylamide gel well. After the electrophoresis, all proteins were transferred onto the polyvinylidene fluoride (PVDF) membrane. Then, the PVDF membrane that contained the target protein was cut and blocked with 5% dry milk for 24 h. Afterwards, the PVDF membrane was washed with PBS for three times, and incubated with Bax, Bcl-2 and cleaved caspase-3 (Sigma Aldrich, St. Louis, MO, USA). Next, the PVDF membrane was incubated with the primary antibodies for 12 h at 4 °C, and washed with PBS for three times. Then, the membrane was incubated with horseradish peroxidase-linked secondary antibodies (Santa Cruz Biotechnology, Santa Cruz, CA. USA) for one hour. Finally, the bands on the PVDF membrane were visualized by enhanced chemiluminescence.

### Apoptosis assay

It has been indicated that AnexA1 promote the apoptosis of inflammatory cells [14]. Therefore, the effect of Ac2-26 on apoptosis-regulated proteins was investigated using the cell model.

The human alveolar epithelial cell line A549 was purchased from the American Type Culture Collection [ATCC], Manassas, CA, USA), and cultured in Dulbecco’s modified Eagle’s medium (DMEM) combined with GlutaMAX (Gibco, Grand Island, NY, USA), 10% foetal bovine serum (FBS, Gibco), penicillin (100 units/ml) and streptomycin (0.1 mg/ml) (Beyotime, Shanghai, China), under 95% air, 5% CO_2_ and 37 °C atmosphere conditions. Then, these cells were washed for three times with serum-deprived DMEM medium, when these cells were cultured to 80% confluence at a density of 10^4^ cells/cm^2^. Afterwards, these cells were administered with PBS, 15 μg/ml of LPS (026:B6, Sigma-Aldrich, St, Louis, MO, USA) [15], LPS + Ac2-26 (0.3 μM), or LPS + Ac2-26 + BOC (10 μM) for four hours, and assigned to the S, A, AA and AAB groups, respectively [7].

### Cell proliferation and viability

The proliferation and viability of the epithelium was evaluated by 3-(4,5-dimethylthiazol-2-yl)-2 5-diphenyl-zolium bromide (MTT) colorimetric assay using specific kits (Sigma, St. Louis, MO, USA). Briefly, the epithelium with a density of 2 × 10^3^ cells/well was plated in 96-well plates. Then, the epithelium was cultured for 48 h. Afterwards, cells in each well were added with MTT (20 μl), and further incubated for four hours. Subsequently, dimethylsulphoxide (150 μl) was injected into the plates, and the plates were further shaken for 10 min. The absorbance of cells was determined using the Multiskan EX (Thermo, Finland) at 570 nm.

The Annexin V-FITC/PI apoptosis detection kit (BestBio, Shanghai, China) was purchased from a commercial company, and used to determine the apoptotic cells through flow cytometry (FACScan, Becton Dickinson, USA). The LPS pre-stimulated epithelium cells were washed with PBS, and cultured in 6-well plates. Then, these cells were suspended in Annexin-binding buffer, and stained with Annexin V-FITC and propidium iodide (PI) for 30 min in a dark room at room temperature. Afterwards, the adherent and floating cells were measured using a flow cytometer (Beckman Coulter, USA) to distinguish the apoptotic cells (Annexin-V positive and PI-negative) from necrotic cells (Annexin-V and PI-positive).

### Statistical analysis

The normality of all data was determined using the Kolmogorov-Smirnov test. Normally distributed data were presented as mean ± standard deviation, while non-normally distributed data were presented in median (IQR). Furthermore, normally distributed data was analyzed by one-way ANOVA, followed by Bonferroni’s *post-hoc* test, while non-normally distributed data was analyzed by non-parametric Friedman test. All statistical analyses were performed using SPSS 19.0 for Windows (SPSS, Inc., USA). *p* < .05 was considered statistically significant.

## Results

### Ac 2-26 improved the alveolocapillary permeability

**Figure 1.**
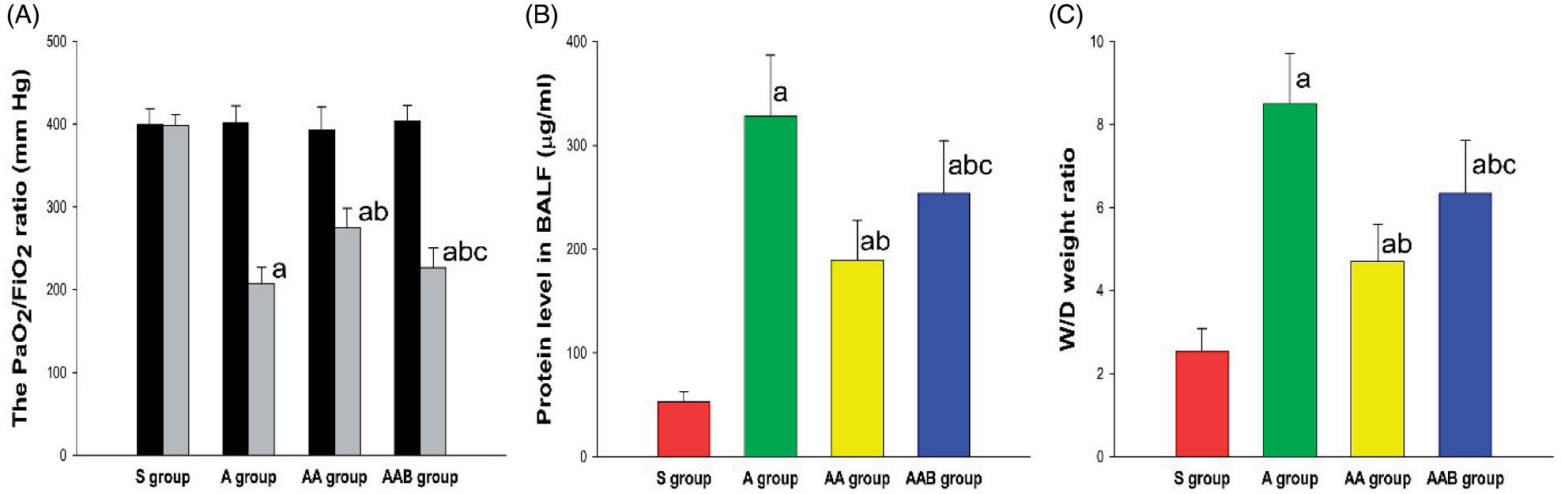
The effect of Ac2-26 on alveolocapillary permeability in ARDS rats. The PaO_2_/FiO_2_ ratio, lung tissue wet/dry ratio, and protein concentration in BALF deteriorated in ARDS rats. Ac2-26 significantly upregulated the PaO_2_/FiO_2_ ratio, and downregulated the wet/dry weight ratio and protein concentration in the AA group. Furthermore, the Ac2-26-mediated improvements in capillary permeability were significantly reversed by BOC-2. ^a^*p* < .05 vs. the S group; ^b^*p* < .05 vs. the A group; ^c^*p* < .05 vs. the AA group (
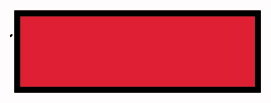
, Sham group; 
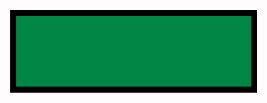
, ARDS group; 
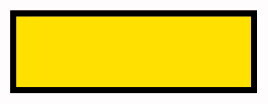
, ARDS/Ac2-26 group; 
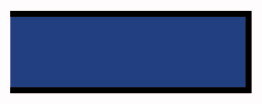
, ARDS/Ac2-26/BOC group).

After 24 h of intratracheal instillation of LPS, the PaO_2_/FiO_2_ ratio significantly decreased in the A, AA and AAB groups. Compared with the A group, the PaO_2_/FiO_2_ ratio increased in the AA group, but the effect of Ac2-26 was lessened by BOC. Similar results were also found in the lung tissue wet/dry weight ratio and protein levels in BALF. Ac2-26 significantly improved the wet/dry weight ratio and protein levels in BALF, but the protection of Ac2-26 was reversed by BOC-2 (Figure 1).

### Ac2-26 reduced the histological injury

**Figure 2.**
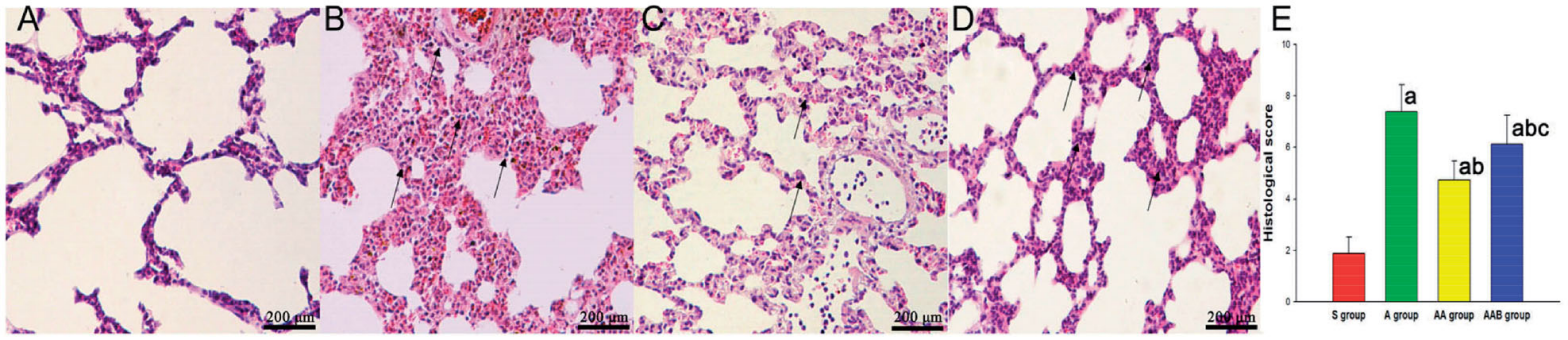
The effect of Ac2-26 on lung histological injury in ARDS. The lung tissue injury was evaluated through H&E staining by independent pathologists, according to Table 1. Ac2-26 significantly reduced lung injury score, while BOC-2 significantly reversed this protection. ^a^*p* < .05 vs. the S group; ^b^*p* < .05 vs. the A group; ^c^*p* < .05 vs. the AA group (
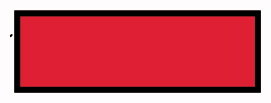
, Sham group; 
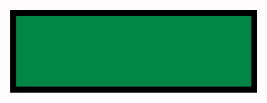
, ARDS group; 
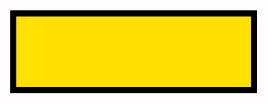
, ARDS/Ac2-26 group; 
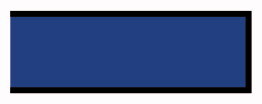
, ARDS/Ac2-26/BOC group).

After 24 h, severe lung tissue damage was observed in rats that received LPS. Compared with rats that received PBS, the Ac2-26 treatment obviously ameliorated the lung injury, which was partially mitigated by BOC. The histological injury score revealed that the Ac2-26 treatment significantly reduced the lung injury, which was partially mitigated by the co-treatment with L-NIO in rats (×200, Figure 2).

### Ac 2-26 inhibited the local and systemic inflammation

**Figure 3.**
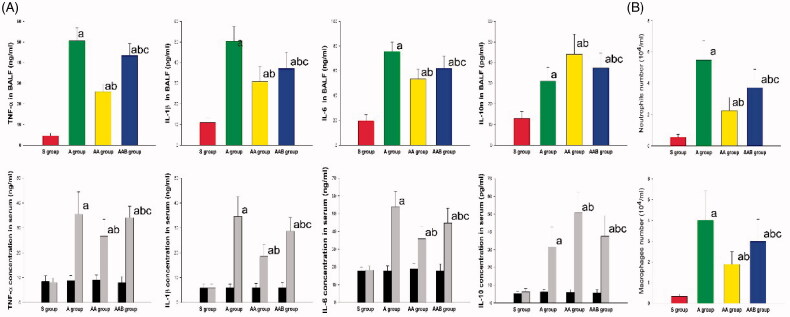
The effect of Ac2-26 on local and systemic inflammation. The levels of TNF-α, IL-1β, IL-6, and IL-10 in serum and BALF were determined by ELISA. The number of macrophages and neutrophils in BALF were also detected. The TNF-α, IL-1β, IL-6, and IL-10 in serum and BALF, and the macrophages and neutrophils in BALF increased in ARDS rats. The TNF-α, IL-1β, IL-6, macrophages, and neutrophils were all reduced by Ac2-26, and the effect of Ac2-26 was reversed by BOC-2. The level of IL-10 increased after the administration of Ac2-26, and this effect was reversed by BOC-2. The data were expressed as mean ± SD. ^a^*p* < .05 vs. the S group; ^b^*p* < .05 vs. the A group; ^c^*p* < .05 vs. the AA group (
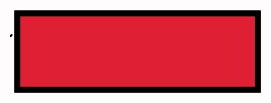
, Sham group; 
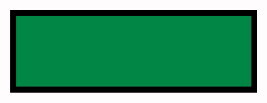
, ARDS group; 
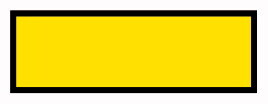
, ARDS/Ac2-26 group; 
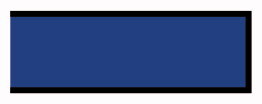
, ARDS/Ac2-26/BOC group; 
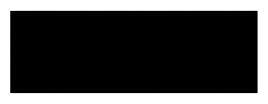
, baseline; 
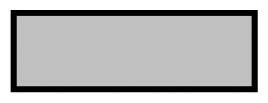
, 24 h after instillation of LPS).

Initially, the inflammatory cell counts and cytokine concentrations of BALF were determined to estimate the effect of Ac 2-26 on local inflammation. After the stimulation of LPS, the macrophages and neutrophils significantly increased in BALF, while the number of inflammatory cells were reduced by Ac2-26. The inhibition of Ac2-26 on the infiltration of inflammatory cells was mitigated by BOC-2 (Figure 3(B)).

Next, TNF-α, IL-1β and IL-6 were detected in BALF and serum to determine the effect of Ac2-26 on local and systemic inflammation. TNF-α, IL-1β, IL-6 and IL-10 were upregulated in BALF and serum in rats that received LPS. Rats that received Ac2-26 had less inflammatory cytokines, when compared to rats without Ac2-26, even though the anti-inflammatory effect of Ac2-26 was lessened by BOC-2. Ac2-26 also induced the upregulation of IL-10 (Figure 3(A)).

### Ac2-26 ameliorated the oxidative stress response

**Figure 4.**
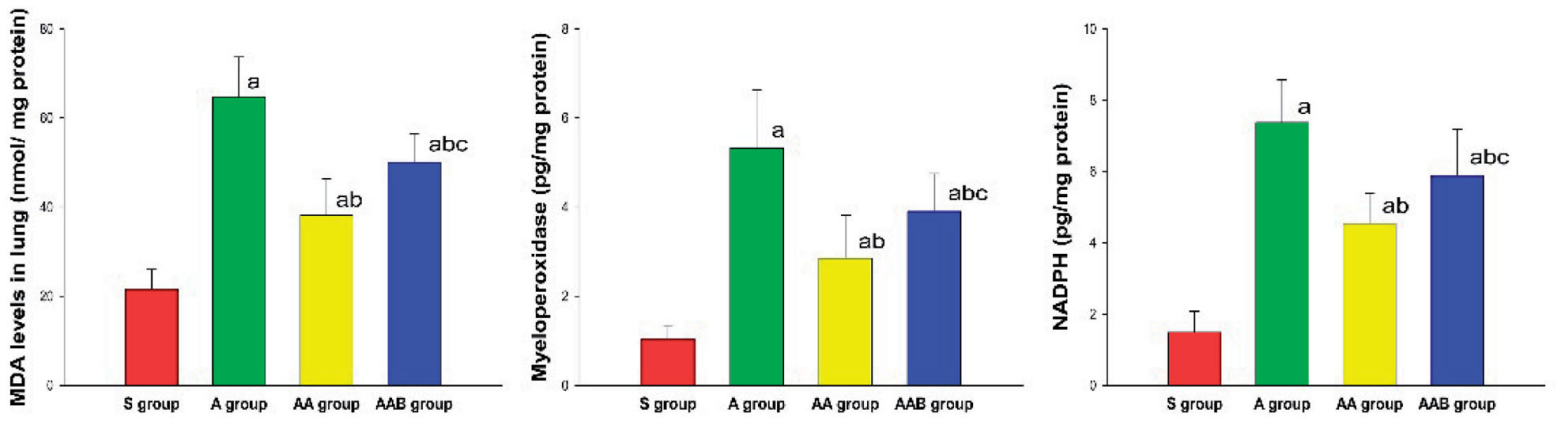
The effect of Ac2-26 on oxidative stress response. The MDA levels, and MPO and NADPH activities significantly increased in lung tissues obtained from ARDS rats, when compared to the S group. The oxidative stress response was significantly decreased by Ac2-26, and the inhibition of Ac2-26 was attenuated by BOC-2. ^a^*p* < 0.05 vs. the S group; ^b^*p* < 0.05 vs. the A group; ^c^*p* < .05 vs. the AA group (
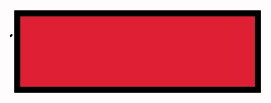
, Sham group; 
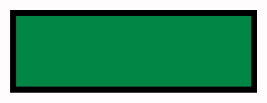
, ARDS group; 
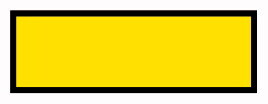
, ARDS/Ac2-26 group; 
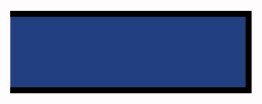
, ARDS/Ac2-26/BOC group).

The activities of MPO and NADPH, and the concentration of MDA in lung tissues were significantly upregulated in ARDS rats, when compared to the S group. These results indicate that ARDS stimulated the oxidative stress response. This response was inhibited by Ac2-26, and the inhibition of Ac2-26 was reversed by BOC-2 (Figure 4).

### Ac2-26 inhibited the epithelium apoptosis and improved the epithelium cell viability

**Figure 5.**
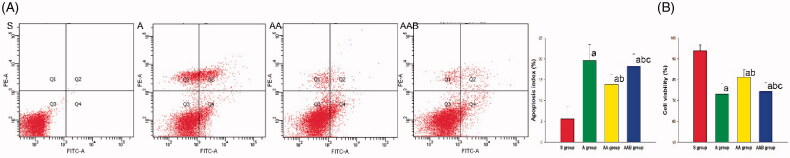
The effect of Ac2-26 on the apoptosis and viability of the epithelium. The apoptosis of the epithelium was estimated by flow cytometry. The apoptosis significantly increased in the A group, but was alleviated by Ac2-26, and this effect was reversed by BOC-2. ^a^*p* < .05 vs. the S group; ^b^*p* < .05 vs. the A group; ^c^*p* < .05 vs. the AA group (
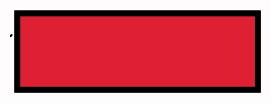
, Sham group; 
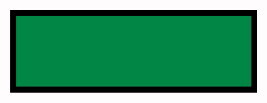
, ARDS group; 
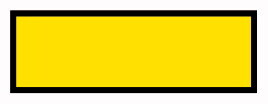
, ARDS/Ac2-26 group; 
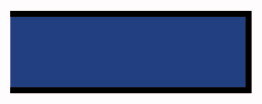
, ARDS/Ac2-26/BOC group).

**Figure 6.**
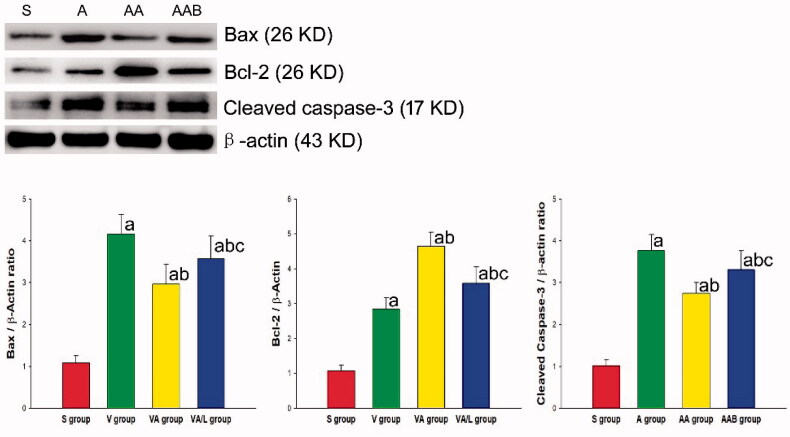
Ac2-26 regulated the apoptosis-related protein expression. The relative expression levels of Bax, BcL-2 and cleaved caspase-3 in the individual groups of cells were determined by western blot and quantification. The data were expressed as mean ± SD for each group, based on three separate experiments. ^a^*p* < 0.05 vs. the S group; ^b^*p* < .05 vs. the A group; ^c^*p* < .05 vs. the AA group (
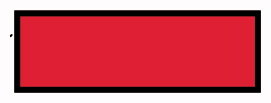
, Sham group; 
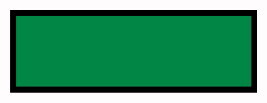
, ARDS group; 
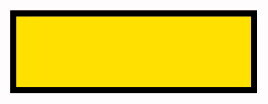
, ARDS/Ac2-26 group; 
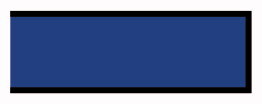
, ARDS/Ac2-26/BOC group).

In order to determine the effect of Ac2-26 on epithelium apoptosis and exclude the influence of other inflammatory cells, the epithelium was solely cultured and stimulated with LPS, and treated with Ac2-26. The results revealed that the treatment with Ac2-26 inhibited the apoptosis of the epithelium induced by LPS (Figure 5(A)). Furthermore, apoptosis-regulated proteins Bax, Bcl-2 and cleaved caspase-3 were also detected in the epithelium. The levels of pro-apoptosis proteins Bax and cleaved caspase-3 were reduced by Ac2-26, but Bcl-2 was upregulated by Ac2-26. The regulation of Ac2-26 on apoptosis and apoptosis proteins was partially reversed by BOC-2 (Figure 6).

The viability of the epithelium was also determined to evaluate the effect of Ac2-26 on the epithelium. The epithelial viability significantly decreased after the treatment with LPS, while this was significantly improved after the treatment with Ac2-26. The improvement of Ac2-26 on epithelium viability was reversed by BOC-2 (Figure 5(B)).

## Discussion

In the present study, it was found that the active peptide of Annexin, Ac2-26, reduced the lung injury, and local and systemic inflammation, improved the lung alveolocapillary permeability, decreased the epithelium apoptosis, and protected the epithelium viability.

It has been reported that ARDS is the major cause of death in critically ill patients [16]. Despite the proceeding of medical treatment and extensive application of lung protective ventilation strategies, the mortality for ARDS patients remain within 27–45% [1]. As an active peptide of Annexin A1, Ac2-26 can ameliorate the lung injury induced by ischaemia/reperfusion injury [6,7]. It has also been shown that Ac2-26 can attenuate the endotoxin-induced lung inflammation [17]. However, the researcher did not explore the mechanism of Ac2-26 on lung injury. In the present study, the ARDS model was prepared to evaluate the effect of Ac2-26 on lung injury, and investigate the possible mechanism of Ac2-26.

In the present study, typical histological injury, the deterioration of PaO_2_/FiO_2_, and the upregulation of proteins in BALF obtained from rats that received LPS were observed. However, the lung injury and capillary permeability were significantly improved by Ac2-26. These results are consistent with previous studies [6,7], indicating that Ac2-26 can alleviate lung injury. In ARDS, after the stimulation of LPS, the damaged epithelium would activate NF-κB [18], and release chemoattractants, such as ICAM-1 and IL-8, which are important factors for chemoattracting inflammatory cells from peripheral blood to the injured lung tissue [19]. The recruited inflammatory cells infiltrate into the injured lung tissue, and produces cytokines into the lung tissue or peripheral blood, further resulting in systemic inflammation [20,21]. In the present study, Ac2-26 significantly reduced the number of macrophages and neutrophils in BALF, and the pro-inflammatory factors in BALF and serum. This result suggests that Ac2-26 attenuates the local and systemic inflammation in ARDS rats. The effect of Ac2-26 on pulmonary inflammation may be due to the inhibition of NF-κB in the epithelium [22]. Furthermore, Ac2-26 also inhibited the release of ICAM-1 and IL-8, and reduced the recruitment and infiltration of inflammatory cells from peripheral blood [23,24]. In contrast to the pro-inflammatory factors, the promotion of Ac2-26 on IL-10 may be another factor. IL-10 can inhibit the injury induced by TNF-α, IL-1β and IL-6, and relieve the lung injury [25]. Compared with rats that only received Ac2-26, the protection of Ac2-26 on inflammation was significantly reversed by BOC-2.

In addition to inflammation, oxidative stress response also plays an important role [26,27] in VILI. During VILI or ARDS, the activated neutrophils convert oxygen into hydrogen peroxide and superoxide anions through NADPH oxidase [28,29]. The ROS not only directly injures endothelial and epithelial cells, but also induces cell apoptosis. In general, MPO, which is enriched in neutrophils, is a marker of neutrophils and the severity of oxidative stress response [30]. MDA, which is the final product of this response, directly indicates the severity of the oxidative stress response [31]. In the present study, it was found that Ac2-26 can significantly reduce the MDA level, and this result suggests that Ac2-26 can inhibit the oxidative stress response. It was hypothesized that this effect was mainly due to the MPO and NADPH pathway [32,33].

In a previous study, apoptosis cells were indicated to participate in the pathology of ARDS induced by LPS [34]. Ac2-26 not only decreases the organic cell apoptosis [9], but also enhances the apoptosis of inflammatory cells to exert its anti-inflammation effect. Therefore, the investigators cultured the epithelium, and administered LPS and Ac2-26, in order to explore the effect of Ac2-26 on epithelium apoptosis. The results indicated that Ac2-26 significantly improved the viability and inhibited the apoptosis of the epithelium. These results demonstrate that Ac2-26 can reduce the epithelium cell apoptosis. In order to explore the mechanism of Ac2-26 on the apoptosis of the epithelium, the protein was extracted to determine the apoptosis regulated protein. Ac2-26 reduced the expression of Bax and cleaved caspase-3, but upregulated Bcl-2 in the epithelium. As an important pro-apoptosis protein, Bax can motivate the cell apoptosis. In contrast to Bax, Bcl-2 can inhibit the activation of Bax. The Bax/Bcl-2 ratio plays a key role in the regulation of apoptosis [35]. Under the effect of Bax, the cleaved caspase-3 would finally cut the DNA, and result in cell apoptosis. In the cell analysis in the present study, the results suggested that Ac2-26 inhibits the apoptosis through the regulation of Bax and Bcl-2. Furthermore, it was also found that the effects of Ac2-26 on cell viability and apoptosis were significantly attenuated by BOC-2.

In order to investigate the mechanism of Ac2-26 on ARDS, BOC-2, an FPR antagonist [7], was administered to rats and the epithelium. FPR is an important protein that regulates inflammation and host defense [22]. The protection and anti-inflammation of AnxA1 in organ injury has been exhibited through FPR [7,36]. The protection of AnxA1 and peptide Ac2-26 was attenuated in Fpr^–/–^ mice [37]. It has also been reported that blocking FPR can mitigate the inhibition effect of Ac2-26 on neutrophils and the shedding of L-selectin [38]. Furthermore, as a key regulator of inflammation, toll-like receptor 4 is activated by endotoxin, and further activates NF-κB, and aggravates the inflammation in ARDS. It had been indicated that the FPR can inhibit the activation of NF-κB, which is the downstream of toll-like receptor 4 [39]. Ac2-26 can reduce the heart infract size and recover the left ventricular function in heart reperfusion injury via the FPR pathway [40,41]. The results of the present study also revealed that the protection of Ac2-26 on ARDS and the damage of the epithelium may be associated with FPR.

## Limitations

The present study had the following limitations: (1) The observation time was only 24 h. In clinic, ARDS is usually treated for several days. In future studies, the investigators will observe the long-term effect and survival of Ac2-26 on ARDS. (2) The effect of Ac2-26 was not observed on other types of alveolar cells, which also participate in the pathology of ARDS. In future studies, the investigators will further investigate the effect of Ac2-26 on type-2 alveolar cells. Furthermore, human epithelial cells were applied, but not rats, to provide experimental evidence for the clinical application of Annexin A1. (3) BOC did not wholly reverse the therapeutic effect of Ac2-26. This result suggests that there may be other possible pathways that participate into the therapy of Ac2-26. The other possible mechanisms of Ac2-26 on ARDS would be further explored in future studies. (4) ARDS rats that received BOC-2 were not enrolled into the study, because BOC-2 shortens the survival time of ARDS rats. Hence, the lung sample and data at 24 h after the injection of endotoxin could not be collected. (5) In the present study, the relationship of FPR and TLR4 was not explored. Although a number of studies have indicated that FPR participates in anti-inflammation *via* inhibiting the adherence of leukocytes to endothelial cells, the effect and mechanism of FPR on TLR4 remains unknown. In future studies, the effect and mechanism of FPR on TLR4 would be investigated.

## Conclusion

The results of the present study suggest that Ac2-26 ameliorates ARDS, and that the protection of Ac2-26 may be associated with FPR. Considering the clinical application of Annexin A1 on lung disease [42], it was speculated that Annexin A1 may be another new therapeutic treatment approach for patients with ARDS.

## Data Availability

The raw data that supports the conclusions of the study will be made available by the authors, without undue reservation, to any qualified researcher.
